# Plant regeneration from leaf mesophyll derived protoplasts of cassava (*Manihot esculenta* Crantz)

**DOI:** 10.1371/journal.pone.0278717

**Published:** 2022-12-01

**Authors:** Asunta Mukami, Bicko Steve Juma, Cecilia Mweu, Mathew Ngugi, Richard Oduor, Wilton Mwema Mbinda

**Affiliations:** 1 Department of Life Sciences, South Eastern Kenya University, Kitui, Kenya; 2 Institute for Biotechnology Research, Jomo Kenyatta University of Agriculture Technology, Nairobi, Kenya; 3 Department of Biochemistry, Microbiology and Biotechnology, Kenyatta University, Nairobi, Kenya; 4 Department of Biochemistry and Biotechnology, Pwani University, Kilifi, Kenya; 5 Pwani University Biosciences Research Centre (PUBReC), Pwani University, Kilifi, Kenya; Lovely Professional University, INDIA

## Abstract

A high yield of isolated protoplast and reliable regeneration system are prerequisite for successful somatic hybridization and genome editing research. However, reproducible plant regeneration from protoplasts remains a bottleneck for many crops, including cassava. We evaluated several factors that influence isolation of viable protoplasts form leaf mesophyll, induction of embryogenic calli, and regeneration of plants in three cassava cultivars; Muchericheri, TMS60444 and Karibuni. A relatively higher protoplast yield was obtained with enzyme mixture containing 5 g/L Macerozyme and 10 g/L cellulase. Muchericheri recorded relatively higher protoplast yield of 20.50±0.50×10^6^ whereas TMS60444 (10.25±0.25×10^6^) had the least protoplast yield in 10 g/L cellulase and 4 g/L cellulase. Freshly isolated protoplast cells were plated on callus induction medium (CIM) solid medium containing MS basal salt, 60 g/L D-glucose, 30 g/L sucrose, B5 vitamins, 100 mg/L myo-inositol, 0.5 mg/L copper sulphate_,_ 100 mg/L casein hydrolysate, 4.55 g/L mannitol, 0.1 g/L MES, 10 mg/L picloram and 3 g/L gelrite to induce protoplast growth and development. The three cultivars reached colony formation but no further development was observed in this culture method. Protoplast growth and development was further evaluated in suspension culture using varying cell densities (1, 2 and 3× 10^5^ p/mL). Development with highest number of minicalli was observed in cell density of 3× 10^5^ p/mL. Minicalli obtained were cultured on CIM supplemented with 10mg/L picloram. Callus induction was observed in all cell densities with the cultivars. Highest somatic embryogenesis was observed in 2× 10^5^ p/ml while no somatic embryogenesis was observed in cell density of 1×10^5^ p/mL. Somatic embryos were matured in EMM medium supplemented with 1 mg/L BAP, 0.02 mg/L NAA and 1.5 mg/L GA_3_ then germinated in hormone free medium for plant regeneration. This protocol which used simple mixture of commercial enzymes is highly reproducible and can be applied in biotechnology research on cassava.

## Introduction

Cassava (*Manihot esculenta* Crantz) is a significant food crop grown worldwide and is considered the most essential root crop for millions of people globally [1]. Cassava is a food security crop due to its resistance to poor soils, drought, unpredictable rainfall, and the ability to postpone tuber harvest until required. In sub-Saharan Africa, it is mostly utilized for alcohol, snacks, and flour. Besides, it is also increasingly used as animal feed and as raw material for different industrial products [2]. The importance of cassava and its enormous potential for genetic improvement makes it a target crop to achieve the United Nations Millennium Development Goals [3]. Cassava production, particularly in sub-Saharan Africa, is below optimal, based on the current yields average which is 20% lower than those attained under ideal growth circumstances [4]. The most prevalent and deadly diseases that impair crop production in sub-Saharan Africa and result in yield losses of up to 90% or crop failure include cassava mosaic disease, cassava brown streak disease, and cassava bacterial blight [5]. Cassava production and utilization is also impeded by short-postharvest shelf life of the storage roots due to post-harvest physiological deterioration (PPD), a physiological and biochemical process that is triggered by the unavoidable physical damage of storage roots during harvesting. PPD is characterized by initial blue/black discoloration and organoleptic changes in the storage roots within 24–72 hr of harvest, which severely renders the storage roots unpalatable and unmarketable [6]. Furthermore, cassava usage is impeded by potentially toxic quantities of cyanogenic glycosides, linamarin, and lotaustralin in all plant organs with exception of the seeds [7] and this demands the need for cassava storage roots to be processed before eating to remove or reduce their levels.

Despite cassava’s immense economic significance and social relevance, not much has been done to develop it in order to address production constraints and conform to customer tastes. Traditional breeding techniques have been used in cassava in the past, but they have had mixed results due to the crop’s high heterozygosity (0.61–0.84%), allochronic blooming, low seed set, and extended breeding cycles [8]. Applications of biotechnological tools in cassava offers an alternative to circumvent the hindrances posed by traditional breeding. Such biotechnological tools include somatic hybridization, genetic engineering and genome editing. These technologies require a reliable and effective plant regeneration system to be initially established. The current available cassava regeneration systems were based on somatic embryogenesis and organogenesis and little efforts, if any, have focused on developing protoplast isolation and regeneration system for African cassava cultivars.

Protoplasts have a great capacity for dedifferentiation, and when grown, they produce cell walls and engage in cell division, enabling the regeneration of the entire plant [9]. Protoplast isolation and regeneration are the key obstacles to all plant studies using them [10, 11]. Hence, investigating the processes of protoplast isolation, culture, culture techniques, colony and callus formation, and regeneration is crucial. The current study presents a dependable approach for isolating protoplasts as well as a potential culture and regeneration technique for Kenyan cassava cultivars.

## Materials and methods

### Plant material and *in vitro* propagation

Three cassava cultivars were used: TMS60444, Muchericheri and Karibuni, and were sourced from Kenya Agricultural and Livestock Research Organization, Gene Bank, Nairobi, Kenya and stocked at Kenyatta University. Stem nodes of 1.27 cm thickness and 1–1.5 cm long containing one axillary bud were isolated from pre-propagated cassava cuttings and surface sterilized by immersion in 70% (v/v) ethanol for 2 min followed by three washes using sterile distilled water. The explants were further sterilized by 15% (v/v) sodium hypochlorite containing a drop of Polysorbate 20 for 10 min. Surface sterilized nodes were rinsed thrice with double distilled water and sprouted aseptically on micropropagation medium (MM) containing MS basal salt with vitamins [12] supplemented with 30% sucrose and 3 g/L gelrite. The medium pH was adjusted to 5.8 before adding 3 g/L gelrite, followed by autoclaving at 121°C for 15 min under 15 kPa. The cultures were incubated at 26±2°C at 16/8 hr photoperiod to induce germination for 3 weeks. The survival and sprouting efficiency for the three cultivars was estimated after 3 weeks of culture.

### Protoplast isolation and purification

**Fig 1:**
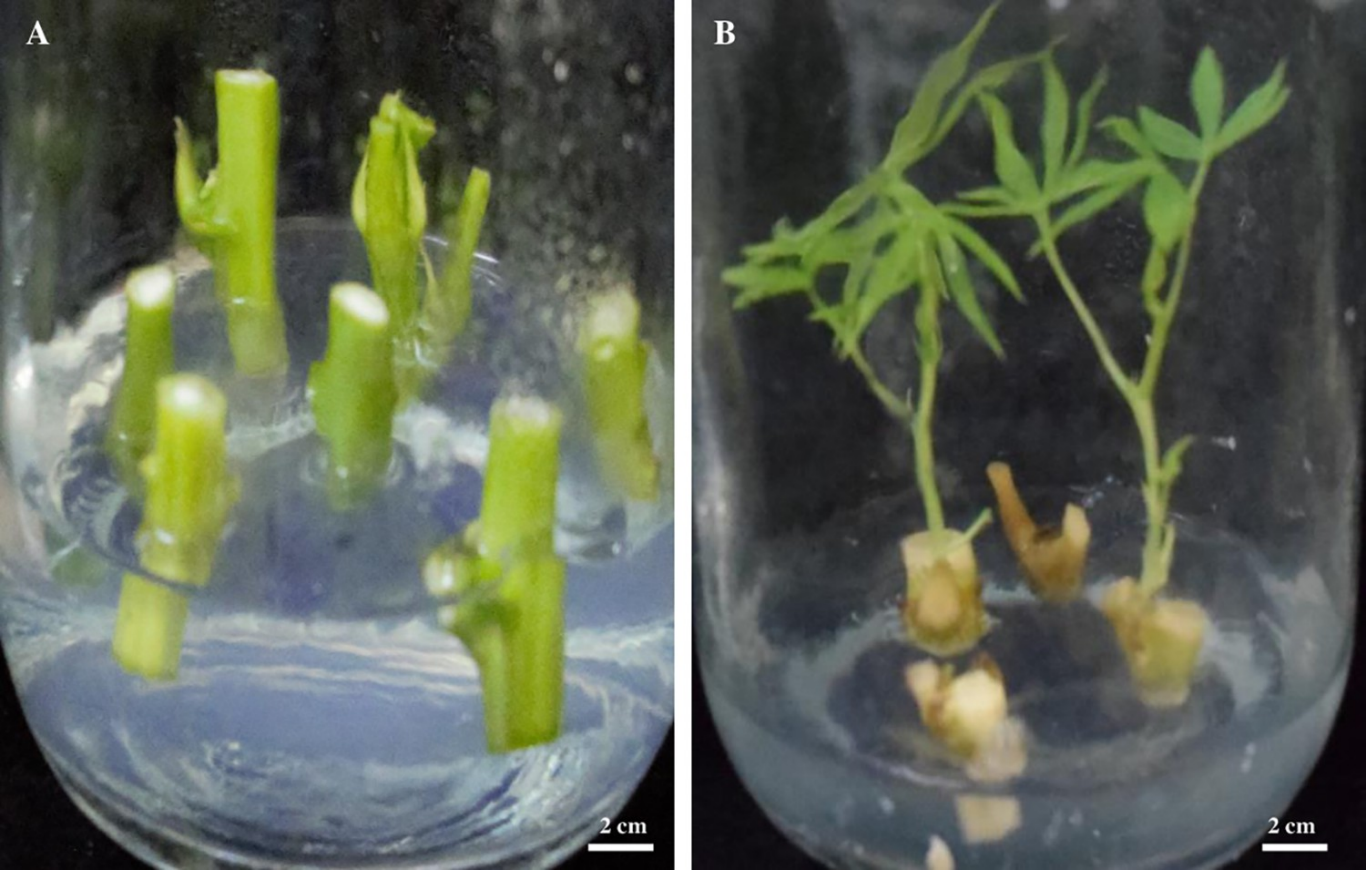
Cassava node propagation. A. cassava nodes with auxiliary buds cultured on MS medium. B. Node germinating on MS medium after 2 weeks culture.

**Fig 2:**
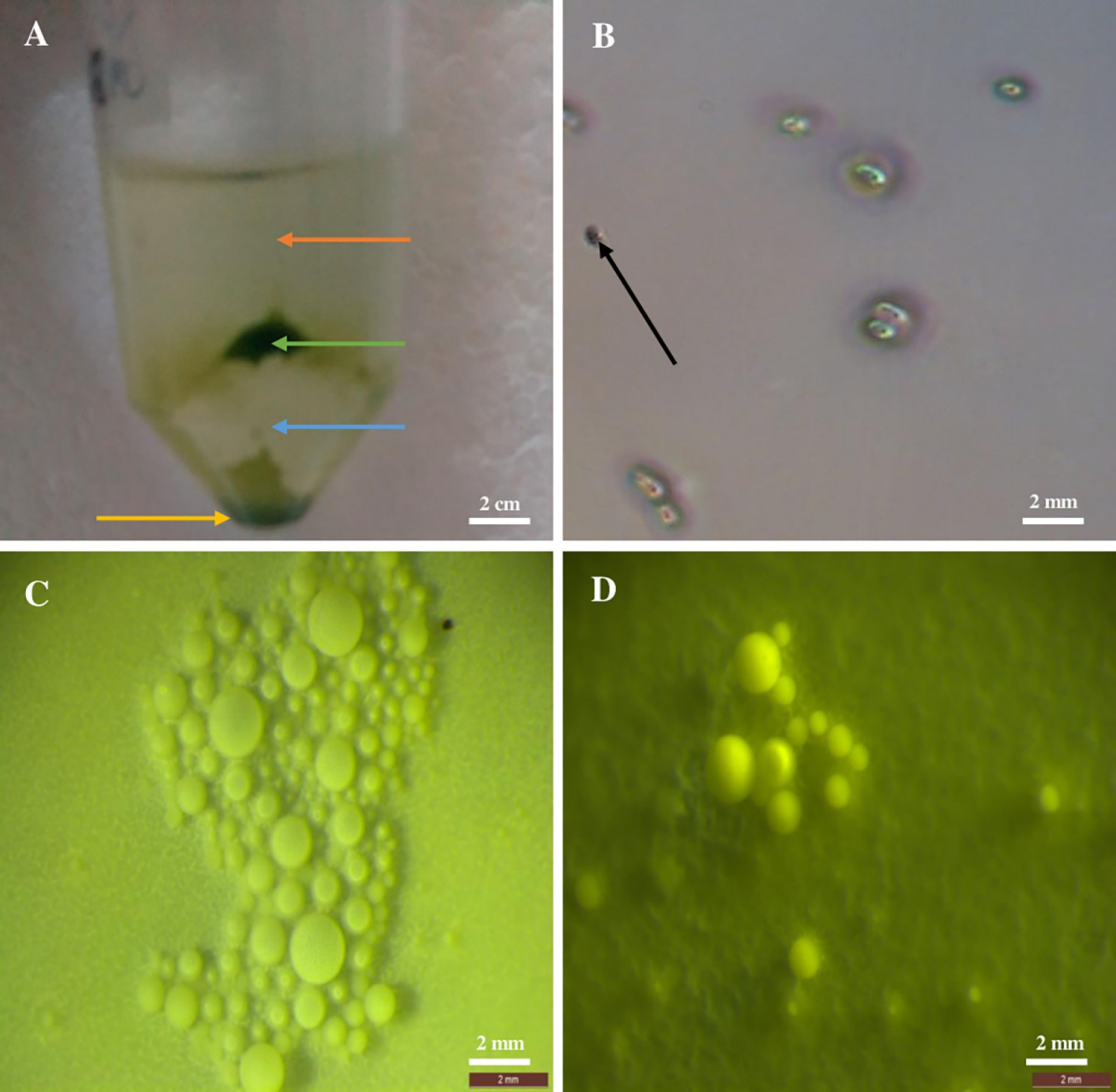
Protoplast solid media culture: **A.** Protoplast gradient centrifugation. Yellow arrow indicates the dead protoplast cells, blue arrow indicates the sucrose solution, green arrow indicate a cushion of living protoplast cells and orange arrow indicates the washing medium. **B.** Protoplast cells under ×100 after Evans-blue staining. Black arrows indicate non-viable cells. **C.** Protoplast growing on solid medium. **D.** Cell clumps/colonies on solid medium.

Protoplast isolation was done using the procedure described by [13] with some modifications. 1 g of fully expanded leaves from *in vitro* plantlets (Fig 1A and 1B) was excised with a sharp scalpel without tissue crushing at the cut site and placed in a Petri-dish (9 cm diameter) with 10 mL of plasmolysis solution containing 0.8 M mannitol, 0.05 M calcium chloride and 0.1% 2-(N-morpholino) ethanesulfonic acid (MES) and left in the dark for 1 hr. The solution pH was adjusted to 5.7 before 0.2 μm microfilter sterilization. The plasmolyzed tissue were then transferred into 10 mL of cell wall digestion solution containing 0.4 M mannitol, 8 mM calcium chloride, 0.1% MES, and the following different enzyme mixture concentrations: 10 g/L Macerozyme (MR), 4 g/L Cellulase (CL) RS-Onozuka; 0.2 g/L MR, 10 g/L CL RS-Onozuka and 5 g/L MR, 10 g/L CL RS-Onozuka. The solution pH was adjusted to 5.7 before 0.2μm microfilter sterilization. The tissue was agitated in dark at 65 revolutions per minute (rpm) for 16 hr. Thereafter, the protoplast suspension was sieved through a 0.75 mm nylon mesh into a beaker. The filtrate was transferred into 50 mL sterile tubes. To determine the best centrifugation speed and time for protoplast collection and purification, the centrifugal speed and time was optimized using a centrifuge with a rotor with 1.5 cm radius, minimum speed of 1000 rpm (16gr) and maximum speed of 6000 rpm (600 gr) The supernatant was discarded and protoplasts pellet resuspended in 4 mL washing solution containing 5 mM glucose, 154 mM sodium chloride, 125 mM calcium chloride, 5 mM potassium chloride and 0.1% 2-(N-Morpholino) ethanesulfonic acid (MES). The solution pH was adjusted to 5.7 before autoclaving. Protoplasts were carefully deposited on 8 mL of 21% sucrose solution followed by centrifugation at 3200 rpm (172 (gr) for 15 min. Protoplast pellets were harvested at the surface of sucrose then resuspended in 15 mL washing solution. The cells were counted in a standard hemocytometer chamber and yield was expressed as the number of protoplasts per gram of fresh weight (g FW) calculated by the formula: Yield = N×5×10^4^×V/ W. Wherein N, is the number of protoplasts obtained by counting; V, the volume of protoplast after dilution, and W, weight of leaf material used for protoplast isolation. Protoplast viability was determined using 1% Evans blue. Blue stained cells were non-viable protoplast cells whereas viable protoplast cells were green (Fig 2B). Protoplast suspensions were centrifuged further at 16 gr for 10 min and the pellets were resuspended in 3 mL of modified protoplast culture medium (PCM) containing MS basal salt, Gamborg B5 vitamins [14] (, 0.4 M mannitol, 0.4 M glucose, 10 mg/L picloram and 0.1% MES [13].

### Protoplast culture and regeneration

**Fig 3:**
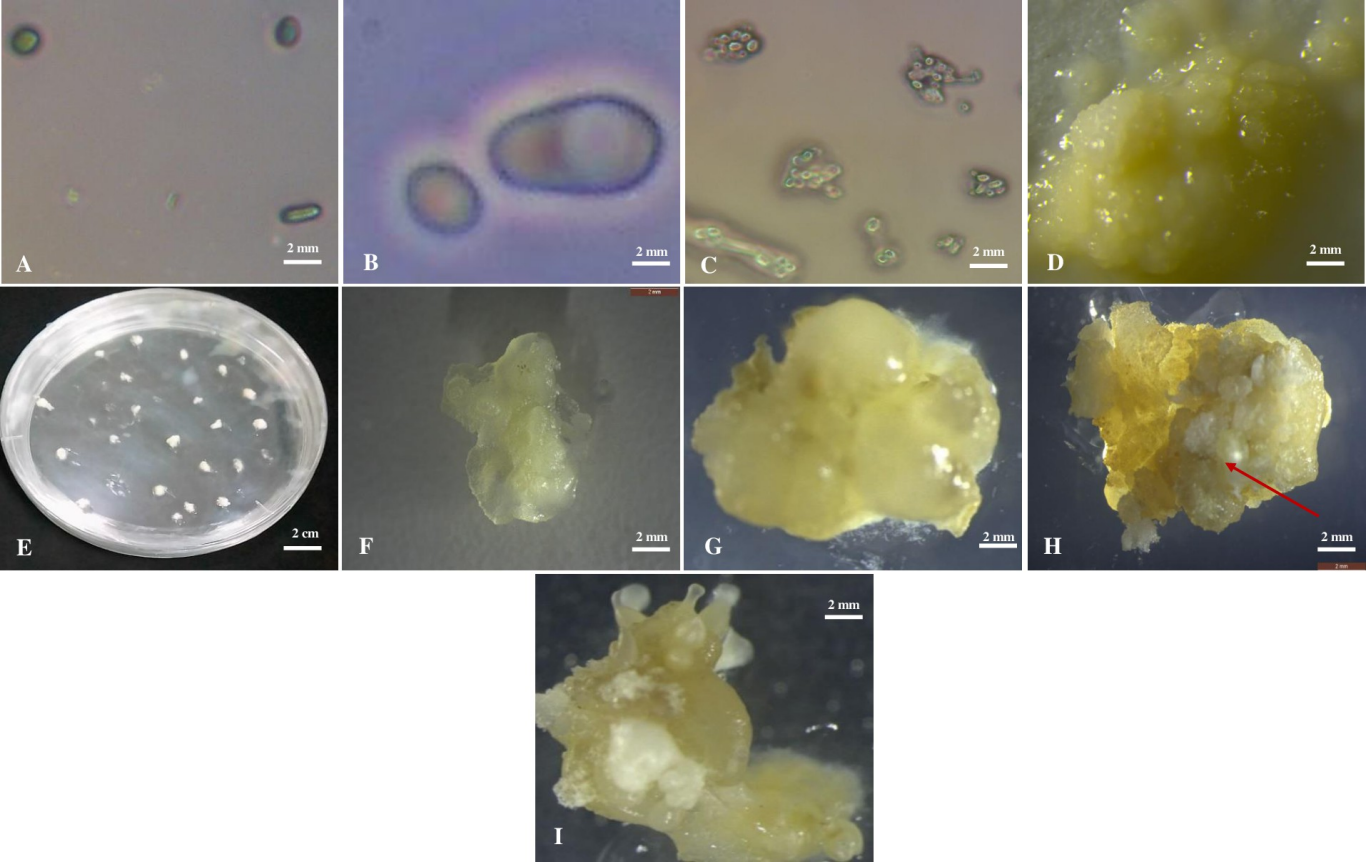
Protoplast suspension culture. **A.** Freshly isolated protoplast. **B.** Dividing **p**rotoplast three days after culture initiation. **C.** Microcalli developing two weeks after culture initiation. **D. M**inicalli 4 weeks after culture initiation. **E.** Minicalli developing on solid medium**. F** Callus obtained from minicalli after 28 days of minicalli culture. **G.** Compact callus. **H.** Callus with somatic embryos after 28 days of culture. Black arrows indicate the developing somatic embryos. **I.** Callus with torpedo shaped somatic embryos.

Two types of culture methods: solid media culture and suspension culture were tested. To determine the effect of plating density on and efficiency of solid medium culture on the formation of cell colonies and microcalli, 100 μL protoplast suspension (of densities 1, 2 and 3× 10^5^ protoplasts/mL) that displayed viability of 90% and above were cultured on freshly prepared solid callus induction medium (CIM) containing MS basal salt, 60 g/L D-glucose, 30 g/L sucrose, B5 vitamins, 100 mg/L myo-inositol, 0.5 mg/L copper sulphate_,_ 100 mg/L casein hydrolysate, 4.55 g/L mannitol, 0.1 g/L MES, 10 mg/L picloram and 3 g/L gelrite. Each culture was replicated ten times in 9 cm Petri dishes. The plates were then sealed and separately incubated in dark at 28°C. Data on cell clumps/colonies was recorded after 4 weeks of culture. After 30 days, the medium containing colonies was cut into blocks of equal sizes and transferred into 30 mL of CIM medium free of gelrite and continuously shaken at 65 rpm in dark. In the second experiment, 100μL of freshly isolated cassava protoplasts were cultured in 5 mL of freshly prepared CIM suspension medium and shaken in the dark at 65 rpm in a rotary shaker at 26±2°C. at densities of (1, 2 and 3× 10^5^ protoplasts/mL) 3 mL of fresh medium containing less glucose (50 g/L, 40 g/L, 30 g/L and finally 20 g/L) was added after every 7 days. After 4 weeks of culture, the minicalli obtained from various cell densities (1, 2 and 3×10^5^ p/mL) (Fig 3D and 3E) were cultured and subculture bi-weekly on freshly prepared CIM free of D-glucose, MES and mannitol to induce the development of calli. The calli obtained were sub-cultured every 14 days on fresh medium for 28 days.

### Maturation of somatic embryos

The calli with globular embryos were induced to form cotyledonary embryos with defined shoot and root apices by sub-culturing the embryogenic calli onto embryo maturation medium (EMM) containing MS basal salt supplemented Gamborg B5 vitamins, 30% sucrose, 0.5 mg/L copper sulphate_,_ 100 mg/L myo-inositol, 100 mg/L casein hydrolysate, 1 mg/L BAP, 0.02 mg/L NAA, 1.5 mg/L GA_3_ and 3 g/L gelrite [15]. The cultures were incubated in dark at 26±2°C. The calli that formed were sub-cultured biweekly for four weeks.

### Desiccation and germination of somatic embryos

In order to remove the phenolic compounds, the greening cotyledonary embryos with defined shoot and root apices were cultured on desiccation medium comprising of EMM supplemented with 0.4 g/L activated charcoal [15]. The number of embryos with shoots were recorded after 7–14 days and transferred to MM. Embryos with stunted shoot growth were sub-cultured in MM supplemented with 0.4 mg/L 6-BAP. The cultures were maintained at 28°C under light for 16/8 hr light/dark photoperiod. The number of rooted plantlets were recorded after one month of incubation. For hardening and acclimatization process, the rooted plantlets were washed with double distilled water to remove medium and thereafter transferred into sterile peat moss in plastic cups (11×15 cm) for hardening for 14 days in glasshouse after which the plants were transferred into soil in pots for growth to maturity in greenhouse.

### Statistics data analysis

The experiment was completely randomized block design with ten replications of five plants for cassava node propagation. For protoplast yield and viability, means were calculated based on two independent isolation per cultivar and experiments repeated thrice. Sixteen microscopic fields per isolation were screened and all cells in the five big hemocytometer squares were analyzed for protoplast yield and viability evaluation. The protoplast culture on solid medium were set up in 9 cm Petri-dishes and 50ml falcon tubes for liquid culture with ten replications per cell density. MS basal medium excluding plant growth regulators and other additives was used as a negative control. Observations for any morphological changes formed on cultures were made weekly. The frequencies of callogenesis and embryogenesis were calculated as the number of callus or embryos formed as a percentage of the total number of explants cultured. The variability of the data was expressed as a mean ± standard error (SE). The data collected were analyzed using one-way analysis of variance (ANOVA) in Minitab statistical computer softwarev.17. Means were separated using the Fisher protected LSD test at confidence level of 95% (p≤0.05).

## Results

### Effects of enzyme combination and concentrations on yield and viability of cassava mesophyll protoplast

Protoplast was successfully isolated in all the combinations of MR and CL. A relatively low yield was recorded in the experiment using 10 g/L Macerozyme and 4 g/L Cellulase. Although this enzyme mixture resulted in the lowest protoplast yield, it did not affect the viability of the cells. Lowering the concentration of macerozyme R-10 and raising the concentration of cellulase R-10 to 1:50 increased the yield of protoplast. (Table 1). However, the yield in this enzyme mixture was relatively lower compared to 5 g/L MR, 10 g/L CL which exhibited higher protoplast yield than other concentrations tested ranging from 20.50 to 10.25x10^6^ protoplast/g FW (Table 1). Muchericheri had a substantially higher response of protoplast yield with 20.50×10^6^ protoplast/g FW. The yield of TMS60444 and Karibuni was not significantly different with change in enzyme combination. However, on close observation TMS60444 produced relatively higher number of protoplasts of 17.00×10^6^ protoplast/g FW than Karibuni which yielded 16.75×10^6^ protoplast/g FW (Table 1).

**Table 1:** Effects of macerozyme and cellulase enzymes combination ratio on yield and viability of cassava protoplast.

	10g/l MR, 4g/l CL		0.2g/l MR, 10g/l CL		5g/l MR, 10g/l CL	
Cultivar	Yield (*10^6^)	Viability (%)	Yield (*10^6^)	Viability (%)	Yield (*10^6^)	Viability (%)
Muchericheri	11.50±0.00^a^	97.24±0.24^a^	16.50±0.50^a^	98.63±0.03^a^	20.50±0.50^a^	98.78±0.34^a^
TMS60444	10.25±0.25^b^	97.37±0.23^a^	15.50±0.00^a^	98.46±0.29^a^	17.00±0.00^b^	98.46±0.31^a^
Karibuni	11.75±0.25^a^	97.96±0.82^a^	16.25±0.25^a^	98.75±0.27^a^	16.75±0.25^b^	97.71±0.44^a^

All protoplast cells were isolated from leaf mesophyll. Yield was calculated as the average of cells in one large hemocytometer square multiplied by dilution factor ×10^4^× original volume/weight of leaf material used and expressed as the number of protoplasts per gram of fresh weight (gfw). Viability was calculated as the percentage number of viable cells from the total number of protoplast (viable and dead). Means (± SE) followed by different letters in each column are significantly different (P≤0.05) using Fishers LSD.

#### Effects of centrifugation speed and time on protoplast purification

The separation of protoplast from digestion solution using a centrifuge with a rotor radius of 1.5cm was achieved when 1000 rpm (16 gr) was used for 10 min. In the subsequent purification process using 21% sucrose, protoplast cells could not separate from the mixture of washing solution and sucrose solution at 1000 rpm (16 gr) irrespective of increased separation time. Therefore, centrifugal speed and time were steadily increased, and at 3200 rpm (172 gr), for 15 min, viable protoplast cells were separated from dead protoplast cells (Fig 2A).

### Effects of plating density and efficiency of CIM solid medium culture on formation of cell colonies and microcalli

After 3 days of protoplast culture in CIM, single cells were observed lodged inside the medium. Cell division was observed from respective single cells identified with an increase in the number of cells after one week of protoplast culture (Fig 2C). Further incubation resulted in cell clump/colony formation in the three cultivars and in all cell densities tested (Fig 2D). With an increase in cell density, a comparatively higher number of cell colonies were observed. The number of cell clumps that developed after 4 weeks of incubation, however, differed between the different cassava cultivars. Statistical analysis of variance showed significant variations in cell colony development among the types. The induction of protoplast growth using a protoplast density of 3× 10^5^ p/mL exhibited a significantly better response of cell colonies formation than other densities tested ranging from 2.10 to 10.20 cell clumps per plate. Karibuni produced the lowest response of 2.10 cell colonies per plate using 1× 10^5^ p/mL compared to Muchericheri which exhibited significantly superior response of cell colonies formation (Table 2). The number of cells per colony varied between 10 and 30 cells. Protoplast cells growing on solid medium were globular in shape (Fig 2C and 2D). Further incubation led to the development of cell clumps (colonies) containing cells of various sizes that seemed lodged in the medium (Fig 2D), making subsequent subcultures challenging. The medium containing the colonies was divided into equal-sized blocks, put into 30mL of liquid CIM, and continually shaken at 65 rpm in the dark to overcome the difficulty of sub-culturing the cell clumps. Despite lengthy incubation, these colonies did not continue to divide to produce the microcalli.

**Table 2:** Effects of cell density on cell colony formation on solid media culture.

Cultivar	1.0× 10^5^cells	2.0× 10^5^ cells	3.0×10^5^ cells
Muchericheri	6.80±0.55^a^	8.10±1.16^a^	10.20±0.98^a^
TMS60444	4.60±0.58^b^	5.90±0.53^b^	5.10±0.75^b^
Karibuni	2.10±0.31^c^	3.30±0.68^c^	4.30±0.42^b^
Control	0.00±0.00^d^	0.00±0.00^d^	0.00±0.00^c^

Protoplast cells were cultured on solid media at varying cell densities. Cell colonies was calculated as the number of clumps containing 10 and more cells per clump. Means (± SE) followed by different letters in each column are significantly different (P≤0.05) using Fishers LSD.

### Effects of cell density on minicalli induction in CIM suspension culture

Culture of freshly isolated viable protoplast (Fig 3A) in CIM suspension culture resulted in cell division after three days of incubation (Fig 3B). The formation of microcalli was seen under a microscope after two weeks of cultivation (Fig 3C). Within 3–4 weeks of culture, the minicalli were visibly detected in all the culture densities (Fig 3D). However, the number of minicalli formed varied across the cassava cultivars and culture densities. Significantly higher response was recorded in culture density of 3×10^5^ p/mL than other densities ranging from 1.2 to 3.6 minicalli. Muchericheri recorded significantly highest number of minicalli while Karibuni recorded the least with 1.20 minicalli at culture density of 1× 10^5^ p/mL (Table 3).

**Table 3:** Effects of cell density on minicalli induction in suspension culture.

Cultivar	1.0× 10^5^ cells	2.0× 10^5^ cells	3.0× 10^5^ cells
Muchericheri	1.60±0.37^a^	2.50±0.34^a^	3.60±0.48^a^
TMS60444	1.50±0.31a	1.90±0.41^ab^	2.60±0.31^b^
Karibuni	1.20±0.25^a^	1.30±0.21^b^	2.50±0.31^b^
Control	0.00±0.00^b^	0.00±0.00^c^	0.00±0.00^c^

Protoplast cells were cultured on liquid media at varying cell densities. Induction efficiency was calculated as the number of minicalli per cell density Means (± SE) followed by different letters in each column are significantly different (P≤0.05) using Fishers LSD.

### Effects of cell density on minicalli development and somatic embryogenic calli induction

Calli development was observed after four weeks of culture of minicalli on freshly prepared CIM free of D-glucose, MES and mannitol (Fig 3F). Minicalli development for the three cassava cultivars responded well to different cell densities tested. Additionally, the calli induction response and the number of calli also varied based on cultivar and cell density. Statistical analysis indicated no significant difference of calli induction in cell densities between 1.0×10^5^ and 3.0×10^5^ p/mL. Induction in 2.0×10^5^ p/mL exhibited significantly better response in calli induction than other cell densities tested ranging from 0.33 to 2.67 calli per cultivar. Muchericheri exhibited the highest response of calli induction, while TMS60444 produced a moderate response. Karibuni produced significantly lowest response of 0.33 calli in cell density of 1.0×10^5^ p/mL (Table 4). The lowest performing cell density was 1.0×10^5^ on TMS60444 and Karibuni with an average number of calli of 0.33±0.33 (Table 4). Similarly, somatic embryos developed on induced calli and the somatic embryogenic calli induction response varied based on cultivar and cell density. Induction in cell density of 1.0×10^5^ produced no response of somatic embryogenesis. The best calli induction response was observed in cell density 2.0×10^5^ with 17.78% (Table 4). Significant reduction of somatic embryogenesis was recorded with increase in cell densities from 2.0×10^5^ p/mL to 3.0×10^5^ p/mL. Calli obtained from culture density 1× 10^5^ p/mL developed into compact calli that did not develop into embryos (Fig 3G). Calli obtained from densities 2× 10^5^ and 3× 10^5^ p/mL developed into both compact calli (that did not develop into embryos) and somatic embryogenic calli (Fig 3H and 3I).

**Table 4:** Effects of cell density on minicalli development and somatic embryogenic calli induction.

		1.0×10^5				2.0×10^5				3.0×10^5		
Cultivar	No. Mini-calli	No. calli		Callus induction efficiency (%)	Somatic embryo induction efficiency (%)	No. calli		Callus induction efficiency (%)	Somatic embryo induction efficiency (%)	No. calli	Callus induction efficiency (%)	Somatic embryo induction efficiency (%)
**Muchericheri**	45	1.00±0.58^a^	6.66±3.85^a^		0.00±0.00^a^	2.67±0.33^a^	17.78±2.22^a^		8.88±2.22^a^	1.33±0.67^a^	8.89±4.44^a^	4.44±2.22^a^
**TMS60444**	45	0.33±0.33^a^	2.22±2.22^a^		0.00±0.00^a^	1.67±0.33^ab^	13.33±3.85^ab^		4.44±2.22^a^	0.33±0.33^a^	2.22±2.22^a^	2.22±2.22^a^
**Karibuni**	45	0.33±0.33^a^	2.22±2.22^a^		0.00±0.00^a^	0.67±0.33^b^	4.44±2.22^b^		2.22±2.22^a^	0.67±0.33^a^	4.44±2.22^a^	0.00±0.00^a^

Minicalli from various cell densities were plated in callus induction media separately. Callus induction efficiency was calculated as the percentage number of calli formed from total number of minicalli cultured. Somatic embryogenic calli induction efficiency was calculated as the percentage number of calli with somatic embryos from total number of calli cultured.

### Somatic embryos maturation, shoot and root induction

**Fig 4:**
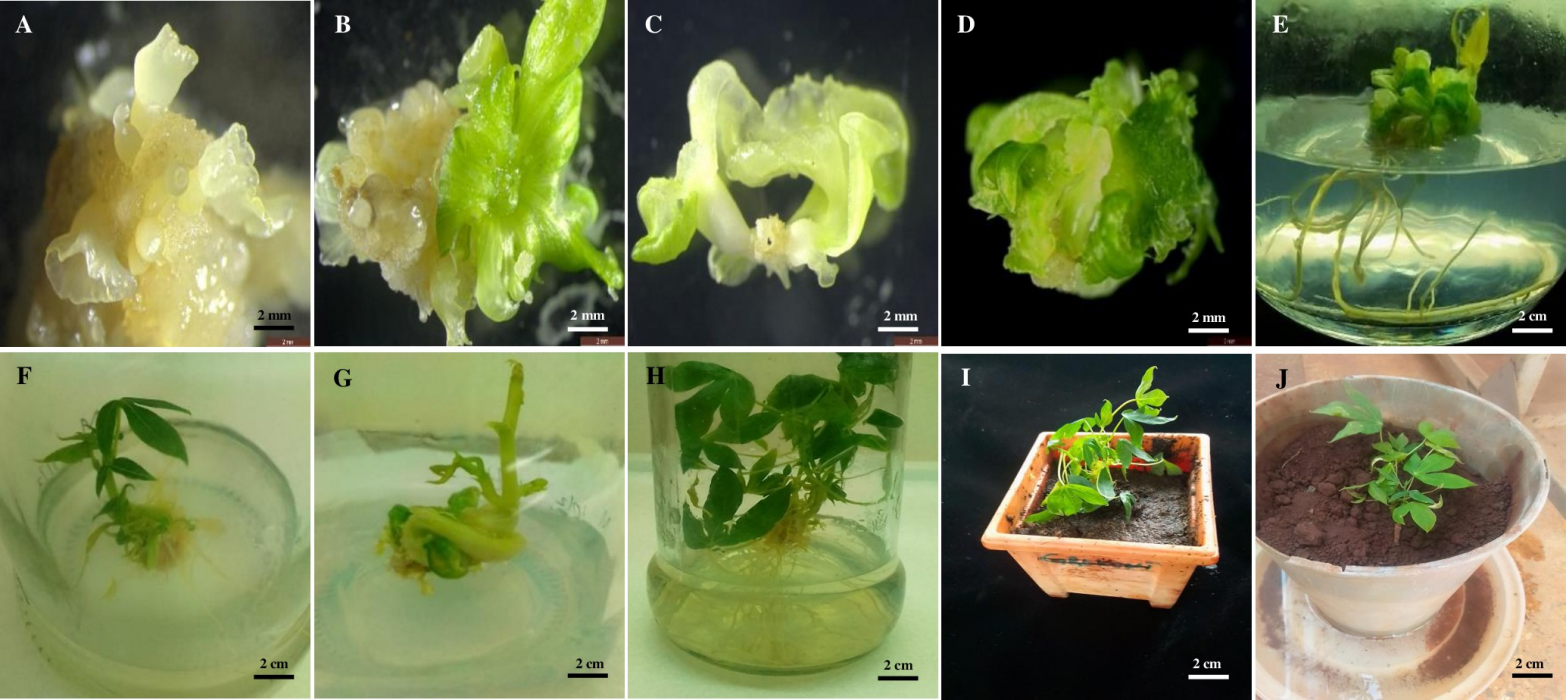
Embryo maturation, shoot and root induction. **A.** cotyledonary embryos after 14 days of culture in maturation media. **B.** Greening cotyledonary embryos after 3 days of culture in desiccation medium. **C.** Greening cotyledonary embryos with defined shoot and root apices. **D.** Wholly greening leafy embryogenic calli. **E.** Rooting plantlet with stunted shoot elongation. **F.** Rooting plantlet with elongating shoot. **G.** Shooting embryogenic calli in 0.4 mg/l BAP. **H.** Rooted plantlet after one month of culture in MM medium. **I.** Hardening plantlet in peat moss. **J**. plantlet growing in soil in the green house.

Maturation of somatic embryos was induced in all cultivars using EMM in dark. After 14 days of culture, the torpedo shaped embryos proliferated to form cotyledonary embryos (Fig 4A). The maturation of somatic embryos to torpedo embryos and finally cotyledonary embryos was cultivar-dependent. Muchericheri recorded the highest number of mature embryos and relatively higher regeneration efficiency than the other two cultivars (Table 5). Embryos were recovered in desiccation medium comprising of EMM supplemented with activated charcoal. Cotyledonary embryos turned green after three days in this medium (Fig 4B). Although after 7–14 days of incubation, some cotyledonary embryos had formed defined shoot and root apices (Fig 4C), some of the embryogenic calli developed large green leaves all over the calli with no defined shoot or root apices (Fig 4D). Initially, shoot and root induction was achieved using MM. Rooting was observed in both embryos without defined root apices (Fig 4E) and those with defined shoot apices after one month of culture (Fig 4F). Although rooting was normal in embryos without defined shoot apices, the shoot growth in these embryos was stunted. Contrary to this observation, shoot elongation was observed in embryos with defined apices (Fig 4F). Shoot proliferation was observed after 14 days of culture (Fig 4G). Root induction and proliferation in both types of somatic embryos was done using hormone free MM for one month (Fig 4H) after which the plantlets were hardened in peat moss for 14 days (Fig 4I), and finally grown to maturity in soil in the greenhouse (Fig 4J).

**Table 5:** Somatic embryo maturation, shoot and root induction.

Cultivar	No. somatic embryogenic calli formed	No. of calli with cotyledonary embryos	No. of regenerated plantlets	Regeneration efficiency
Muchericheri	8	8	3	37.5%
TMS 60444	3	3	1	33%
Karibuni	1	0	0	0%

Calli with somatic embryos were matured in EMM then recovered in EMM supplemented with activated charcoal. Somatic embryogenic calli with defined shoots were rooted in MM medium. Regeneration efficiency was calculated as the percentage of number of regenerated plants from total number of somatic embryogenic calli induced.

## Discussion

The use of protoplast is currently increasing especially with respect to new plant breeding techniques and new genomics research involving single cell. Protoplast isolation and culture have been studied in cassava [16–22]. Although successful shoot regeneration has been reported in callus derived protoplast using model cassava TMS 60444 [19, 20, 22], to-date, no study has reported any successful shoot regeneration of cassava mesophyll protoplast. Our study therefore presents the first reported work on cassava plant regeneration from leaf mesophyll derived protoplasts. Cassava mesophyll protoplast extraction efficiency of 4.4×10^7^ protoplast/ g FW leaf and viability (92.6%) was achieved by [21], which is different from the findings of the previous research in which values of 5.6×10^6^ protoplast /g FW [16] and 1.95×10^7^ protoplast/ g/ FW, 85±2% [16] were obtained. In this study, we factored several conditions in protoplast isolation previously reported in *Arabidopsis thaliana* [13] (and cassava to develop a reproducible, time-saving and efficient method for protoplast isolation and culture. As such, we changed the leaf material preparation, composition of digestion solution and centrifugal speed and time for protoplast collection and purification as well as the composition of the medium. Protoplast are osmotically sensitive; therefore, plasmolysis prior enzymatic digestion of explant helps reduce cytoplasmic damage. Osmotic pressure protective agents can maintain the balance of interior and exterior osmotic pressure of the protoplasts to prevent lysing. The most used inorganic salts are KCl, NaCl, CaCl_2_ or non-metabolizable sugars or sugar derivatives such as mannitol, sucrose or sorbitol or their combination [23]. Previously, leaf pre-treatment in cassava was performed using 0.5 M mannitol and reported a relatively lower protoplast yield (1.95×10^7^ protoplast/ g/ FW) and viability of 85±2% [17], compared to our work which presents yield of 20.50×10^6^ protoplast /g FW and 98.78% viability rate when combination of 0.8 M mannitol and 0.05 M CaCl_2_ were used in leaf pre-treatment. Calcium chloride usually is an ionic substance that has often been combined with non-ionic osmoticum [24]. Its’ use in the range of 2 to 10 mM has been shown to maintain membrane integrity [25]. The inclusion of 10 mM CaCl_2_ into enzyme solution containing 0.6 M mannitol showed better protoplast yield in *P*. *angulata* [26]. In our study, 0.8M mannitol was combined with 0.05 M CaCl_2_ in plasmolysis solution while 0.4 M mannitol was combined with 8 mM CaCl_2_ in enzyme solution. This combination may have contributed to better yields and viability. However, more work should be done with different inorganic salts and sugars combinations.

In protoplast isolation process, the concentration of digestion enzyme mixtures and digestion time directly affect the efficiency and viability of protoplast. To increase protoplast release, a longer enzymolysis time is beneficial. However, prolonged enzymolysis could lead to protoplast breakdown and subsequent decrease in protoplast viability [27]. [17] established a protocol for cassava mesophyll protoplast isolation with an enzyme mixture for 16 hr. In another study [21], also reported both high yield (4. 4×10^7^ protoplast/g FW) and viability of 92.6% of protoplast at 16 hr. In the present study, and guided by our results; yield (20.50×10^6^) protoplast /g FW and viability (98.78%) and results from the two reports, we maintained the enzymolysis time at 16 hr. The combination of enzyme solution influenced protoplast extraction efficiency. Protoplast yield increased with increase in cellulase in the enzyme mixture. The enzyme mixture containing 5 g/L Macerozyme R-10 and 10g/L Cellulase R-10 was the best mixture in terms of yield and viability of protoplast cells in the three cultivars. The protoplast yield reported here is relatively lower than the previously reported yield in cassava mesophyll [21]. The difference could be attributed to the differences in composition of digestion solution, amount of explant used and possibly the cassava cultivars used in the study. However, the high yielding viable protoplast obtained in our study were comparable to values reported for rice, 1.0×10^7^ protoplast/g FW, 94.3% [28] and Cymbidium, 2.5×10^7^ protoplast/g FW, 92.1% [29]. The cultivar-dependent variations in protoplast yield among the cassava cultivars that we observed were also noted in previous studies for dicot cereal plant [30]. Our work obtained high protoplast viability, suggesting that the digestion solution characterized by the presence of combination of mannitol and calcium ions is well adapted to cassava protoplast.

Protoplast purification is an essential process that can affect the yield and viability of protoplast cells. Establishment of appropriate protoplast purification system can help overcome poor recoveries and possible protoplast damages that affect division. Previously, an array of centrifugal speed and time have been reported in purification of cassava mesophyll protoplasts. For example, [16] used 800 g for 20 mins, while [17, 21] reported 80 gr for 10 min and 3 min, respectively. We optimized the centrifugal speed and time for protoplast collection and purification. From our finding, centrifugal speed of 16 gr for 10 min was minimum speed for collection of protoplast cells from digestion solution whereas a centrifugal speed of 172 gr for 15 min was best for separation of live protoplast cells from dead cells in a mixture of washing solution and 21% sucrose solution. Previously [22], performed protoplast purification in cassava using 26% sucrose, which is slightly higher than our conditions. Similar results have been reported in potato protoplast [31]. Sucrose gradient has been reported to secure viable protoplast cells by selectively removing damaged protoplasts and other cellular debris [32].

Cassava mesophyll protoplast development has previously been achieved in semi-solid medium [17] and solid medium [16, 19]. However, no successful plant regeneration was in these studies. In the present study, three cell densities (1.0×10^5^ p/mL, 2.0×10^5^ p/mL and 3×10^5^ p/mL) were tested in both solid and liquid culture systems. Despite the development of microcolonies, the failure of microcolonies to undergo division to form microcallus in solid medium could be due to improper aeration and local accumulation of toxic substances released from dying cells hence inhibiting growth of neighbouring cells [33]. In liquid culture system, the genotype dependent minicalli formation was observed in all tested cell densities. Attaining low callus induction efficiency and low somatic embryogenesis in both 1.0×10^5^ p/mL and 3×10^5^ p/mL cell densities and higher callus induction and somatic embryogenesis in 2.0×10^5^ p/mL implied that 2.0×10^5^ p/mL was the optimal cassava mesophyll protoplast culture density in terms of calli and somatic embryogenic calli induction. Somatic embryogenesis is a process in which somatic cells in a culture redifferentiate and give rise to cells that can form somatic embryos [34] with the occurrence of cell-cell interactions [35]. Previous studies have shown that cells cultured at low cell densities cannot form somatic embryos while cells cultured at high density or at a low density in presence of a cell-free growth medium preconditioned by high-density suspension can undergo somatic embryogenesis [36–38]. However, in this study, higher cell densities of cassava protoplast were unfavorable for somatic embryogenesis which may be due to competition for nutrients or release of inhibitors. Similar results were reported in carrot where somatic embryogenesis was strongly inhibited in high cell density cultures due to conditioning factors into culture medium from cell during culture [33]. Our results also compare with the finding of [39] who reported that increasing the leaf mesophyll protoplast plating density in *Malus domestica ‘*Anna’ from 0.5×10^5^ p/mL to 2.0×10^5^ p/mL resulted in enhanced protoplast development. Beyond the range, the development was adversely affected.

Previous studies failed to regenerate cassava mesophyll protoplast. In most cases only callus proliferation with either root [17, 18] or shoot [16] (were obtained. Notably, the three studies omitted the embryo maturation and recovery stage before shoot and root induction. Maturation of somatic embryos is crucial as it leads to development of embryos at globular stage into green cotyledonary embryos with defined shoot and root apices. In the current study, 10 mg/L picloram previously reported to induce callus and somatic embryogenesis in cassava leaves [15] was used for cassava mesophyll protoplast callus induction and somatic embryogenesis. Additionally, somatic embryos were matured using 1 mg/L BAP, 0.02 mg/L NAA and 1.5 mg/L GA_3_. The low frequency of embryo conversion into plantlet has been observed in numerous systems in spite of high number of somatic embryos produced. In such cases, gibberellic acid (GA_3_) is frequently used to stimulate embryo conversion and improve efficiency of plant regeneration [40]. In this study, the use of high concentrations of GA_3_ compared to the pre-established cassava mesophyll protoplast regeneration methods may have increased plant recovery rates. Increase plant recovery rates have also been previously reported with increase in concentration of GA_3_ [15].

The excessive production of polyphenols during initial stages of culture has been shown to cause browning and eventual death of tissues. This phenomenon was reported in the pre-established cassava mesophyll protoplast culture [18]. Therefore, polyphenols should be eliminated from *in vitro* environment by use of activated charcoal in order to increase explant survival and organogenesis [41]. We used 0.4 g/L activated charcoal to remove phenolic compounds from the cotyledonary embryos. The use of activated charcoal in this study may have increased plant recovery rates attributed to the absorption of auxins that had been used in callus induction medium along with polyphenols produced by the callus.

Cassava leaf mesophyll protoplast regeneration efficiency has not been reported. However, regeneration efficiency of callus derived protoplast in cassava cultivar TMS 60444 has been known to be 18%, 24% and 30% as reported by [19, 20, 22] respectively. In these studies, the plants were regenerated within 6–7 months after protoplast culture initiation. In the present study, cassava mesophyll protoplast derived plants were regenerated within 5 months after culture initiation with a relatively higher regeneration efficiency of 37.5% for cultivar Muchericheri and 33% for TMS60444. Our work therefore presents an improved plant regeneration from leaf mesophyll derived protoplasts of cassava, which could be applied in various biotechnology studies on cassava.

## Conclusion

This is the first account of the cassava plant being successfully regenerated from protoplast derived from leaf mesophyll. A dependable and repeatable cassava protoplast separation and culture technique using leaf mesophyll as the explant has been described in this study. The study also offers a comparison of protoplast genesis and growth in cultures on solid media and liquid/suspension medium. The results reported here demonstrate the impact of protoplast cell densities and choice of culture method on protoplast growth among the studied cultivars. Cassava cultivar Muchericheri recorded higher protoplast cell yield, cell colony, minicalli, calli and somatic embryogenic calli formation compared to other two cultivars. Protoplast cell density of 2.0×105 p/mL provided higher number of somatic embryogenic calli induction when compared to other cell densities. Muchericheri and TMS60444 cultivars displayed considerable potential protoplast growth and regenerability and therefore potentially useful for protoplast fusion and hybridization, single cell cloning and genetic manipulation. Further investigations on solid medium culture conditions for cassava protoplast need to be explored. Further, more work should be done with different combinations of the inorganic salts and sugars to establish the best combination regime.
